# Prevalence of gastroparesis in diabetic patients: a systematic review and meta-analysis

**DOI:** 10.1038/s41598-023-41112-6

**Published:** 2023-08-28

**Authors:** Lianxin Li, Luyao Wang, Ruolan Long, Linrui Song, Rensong Yue

**Affiliations:** Hospital of Chengdu University of Traditional Chinese Medicine, Chengdu, China

**Keywords:** Endocrine system and metabolic diseases, Endocrinology, Gastroenterology

## Abstract

Although there was no significant heterogeneity in the meta-publication, sensitivity analyses revealed significant heterogeneity. Overall, the prevalence was higher in women (N = 6, R = 4.6%, 95% CI 3.1%, 6.0%, and I^2^ = 99.8%) than in men (N = 6, R = 3.4%, 95% CI 2.0%, 4.7%, and I^2^ = 99.6the %); prevalence of type 2 diabetes (N = 9, R = 12.5%, 95% CI 7.7%, 17.3%, and I^2^ = 95.4%) was higher than type 1 diabetes (N = 7, R = 8.3%, 95% CI 6.4%, 10.2%, and I^2^ = 93.6%); the prevalence of DGP was slightly lower in DM patients aged over 60 years (N = 6, R = 5.5%, 95% CI 3.3%, 7.7%, and I^2^ = 99.9%) compared to patients under 60 years of age (N = 12, R = 15.8%, 95% CI 11 15.8%, 95% CI 11.4%, 20.2%, and I^2^ = 88.3%). In conclusion, our findings indicate that the combined estimated prevalence of gastroparesis in diabetic patients is 9.3%. However, the sensitivity of the results is high, the robustness is low, and there are significant bias factors. The subgroup analysis revealed that the prevalence of DM-DGP is associated with factors such as gender, diabetes staging, age, and study method.

## Preface

Diabetes and its complications are now a major global health concern. According to the 10th edition of the International Diabetes Federation's (IDF) Global Diabetes Map^1^, published in 2021, the prevalence of diabetes is 10.5% (1 in every 10 people has diabetes); approximately 643 million (11.3%) adults will have diabetes by 2030, rising to 783 million (12.2%) by 2045.; global diabetes accounts for at least $966 billion in health expenditures, a 316% increase over the last 15 years. According to the Global Diabetes Map, China has the highest prevalence of diabetes patients and undiagnosed diabetes and second in global diabetes health expenditure, indicating that the diabetes situation in China remains critical. Therefore, effective intervention strategies and policies are urgently needed to halt the rise in the number of diabetics. Type 2 diabetes mellitus (T2DM) accounts for approximately 90–95% of the total diabetes population. People with diabetes are always hyperglycaemic because of insulin resistance or insufficient insulin secretion. This results in dysfunction and chronic damage to blood vessels, the brain, nerves, as well as other tissues and organs of the body. As a result, most diabetic patients have gastrointestinal dysfunction in the early or late stages of the disease^2^.

Diabetic gastroparesis (DGP), first described by Kassander in 1958^3^, is a chronic neuromuscular disorder of the upper gastrointestinal tract characterised by impaired gastric motility and delayed gastric emptying^4^. The majority of patients have atypical or no symptoms, and many do not experience significant discomfort, making the disease easy to overlook by patients and clinicians. However, delayed gastric emptying is evident on ancillary tests. Furthermore, the prevalence of diabetic gastroparesis is increasing year by year^5^. Foreign literature suggests^6–8^ that 50–76% of diabetic patients with a long history of the disease have abnormal digestive tract dynamics, while the prevalence of diabetic gastroparesis is unknown due to factors such as trial design, diagnostic basis, sample size, and population differentiation. The treatment of DGP^9^ is based on symptomatic treatment such as gastroprokinetic drugs^10^, gastric electrical stimulation and endoscopic therapy, but the long-term results are not very satisfactory and the quality of life of patients is seriously affected. The latest systematic review and network meta-analysis highlights the paucity of efficacious drugs for the treatment of gastroparesis^11^.

The epidemiological findings show that^12^, while DGP does not affect the life expectancy of diabetic patients, it can affect their digestion and absorption of medication, aggravating their glucose metabolism and making their condition difficult to control, sometimes leading to serious consequences and a significant reduction in their quality of life^13^, as well as imposing a heavy economic burden on their families and society^14^. The significance of DGP in the progression of diabetes should be addressed. Therefore, this meta-analysis was conducted to provide a comprehensive analysis of DGP prevalence in DM around the world to shape healthcare policy.

## Materials and methods

### Search strategy

The protocol of this review was registered at PROSPERO (No. CRD42023389624). We searched the Zhiwang, Wanfang, Vipshop, PubMed, Web of Science, Cochrane Library, and Embase databases until 1 October 2022 for relevant articles. Additional relevant literature was found in the reference lists of the identified articles. The terms used in the search are available in the Appendix.

### Criteria for selecting and enrolling in studies

Two reviewers independently reviewed titles and abstracts discovered via electronic searches to select potentially relevant studies. When the following eligibility criteria were met, the full text of the article was downloaded:

1. Observational studies (cross-sectional, case–control, and cohort studies).
2. A diagnosis of DM can be confirmed through medical records, self-reports, or by a clinician.
3. Gastroparesis diagnosed by the clinician or by using the gold standard gastric emptying scintigraphy (GES), as well as alternative methods or other validated tools, including stable isotope GE breath testing (GEBT), the wireless motility capsule (WMC), and functional ultrasound^15^.
4. The reported recent or lifetime occurrence of gastroparesis in DM patients.

We excluded: (1) studies on functional dyspepsia, (2) studies where the necessary information was not available even after contacting the authors, and (3) unclear methodological and observational studies.

### Data extraction and quality assessment

The following information was extracted independently by both individuals using data extraction forms relating to studies that met the inclusion criteria: prevalence of DM-DGP, country, year of publication, author name, sample size, number of patients with DGP, number of men and women with DGP (if available), age range or mean, and tool for identifying DGP. Reviewers assessed study quality and bias risk using STROBE (Strengthening the Reporting of Observational Studies in Epidemiology) and PRISMA guidelines. In certain cases, the senior reviewer was able to clarify any ambiguity or disagreement between reviewers.

### Statistical analysis

The primary outcome of the study was the prevalence (*p*%) of DM-DGP. We performed a meta-analysis comparing the prevalence (*p*%) and confidence intervals (CI) for each case. STATA 17.0 uses the “Metan and Metareg procedure” to analyze all data. Meta-regression identified potential heterogeneous sources. We used the I^2^ metric to assess between-study heterogeneity and random effects (if I^2^ > 50% or Chi-Square Test *p* < 0.05) for Cochran’s Q analyses. I^2^ values of 25%, 50%, and 75% indicate low, medium, and high levels of heterogeneity between studies, respectively. In the model, a *p*-value < 0.05 was considered significant. Sensitivity analyses were conducted by omitting one study at a time from the impact analysis. Egger’s test was used to assess the article’s publication bias.

### Ethical approval

This article does not contain any studies with human participants or animals performed by any of the authors.

## Results

### Search results and study characteristics

For the first time, the literature review screened 1171 articles based on title/abstract (PubMed n = 951, Embase n = 26, Web of Science n = 115, Cochrane Library n = 25, Zhiwang n = 9, Vipshop n = 2, and Wanfang n = 3). Duplicates were removed (n = 3) and 1,168 articles were obtained and reviewed independently by two reviewers.

**Figure 1:**
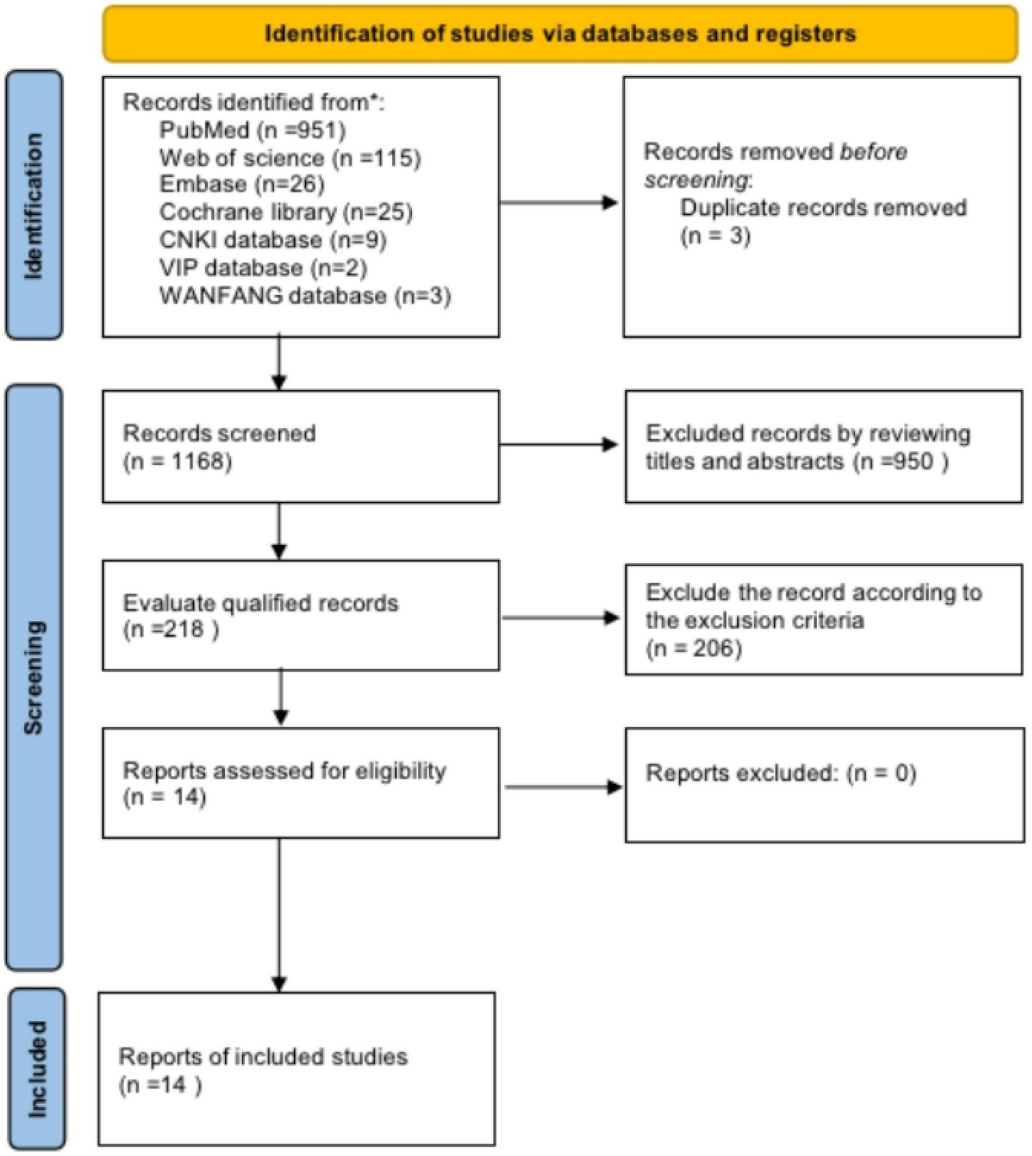
Literature screening process and results.

Finally, a total of 14 articles were included in the meta-analysis, including 12 cross-sectional studies, one cohort study, and one case–control study. Figure 1 shows the flowchart of study selection, while Table 1 shows the characteristics of included studies. A total of 3,200,177 participants with DM were included in the meta-analysis, with 50,833 of them having gastroparesis. Because the diagnostic criteria for DGP have not been standardised and its prevalence varies significantly in national and international reports, the main modalities currently used to diagnose DGP are the Gastroparesis Cardinal Symptom Index (GCSI)^16^ and GES^17^. The GCSI scale total score is a more reliable tool for assessing early gastroparesis, with a significant relationship found between the severity of symptoms assessed by the clinician and the GCSI total score^18^. For the gastric emptying rate test, the main methods include ultrasound, radionuclide, and breath tests. Ultrasound testing does not provide a valid and accurate test for solid food emptying. Radionuclides are expensive and radiologically harmful, but they are often used in scientific research. The breath test is non-radioactive and non-invasive, but its sensitivity and specificity are only about 80%. However, it can be used as a clinical screening test to rule out abnormal gastric motility. Therefore, if a diabetic patient exhibits symptoms such as upper abdominal distension and discomfort, acid reflux, belching, nausea, vomiting, or difficulty controlling blood glucose, an appropriate gastric emptying rate test should be performed promptly to confirm the diagnosis^19^. Type 1 or 2 diabetes is diagnosed by the following criteria: American Diabetes Association (ADA) criteria, World Health Organization (WHO) criteria, and self-report and medical history as determined by the American Diabetes Association.

**Table 1:** Basic characteristics of the included studies.

ID	Inclusion in the study	Region	Survey time	Average age	(cases)	Total sample size	Prevalence (%)	DM typing	M	W	D
1	Aleppo^20^	USA	2010–9 to 2012–8	65	340	7107	4.8%	1	173/4321	167/2786	Gastric emptying scintillation scan
2	Rakan^21^	Saudi Arabia	–	53.47	15	147	10.08%	2	5/51	11/96	GCSI
3	AlOlaiw i^22^	Saudi Arabia	2017–4 to 2018–3	55.26	25	400	6.20%	2	5/175	20/225	GCSI
4	Andersen^23^	Denmark	–	50.40	102	765	13.30%	1	9.73%	9.86%	GCSI
5	Kojecky^24^	Australia	–	62.3	26	147	17.70%	1			GCSI
6	Sfarti^25^	Romania	2008–9 to 2009–2	49.5	26	69	33.7%	1			Gastric emptying scintillation scan
7	Aslam^26^	USA	(1999–2014)	62.70	11,470	249,930	4.59%	1			Gastric emptying scintillation scan
8	Aslam^26^	USA	(1999–2014)	69.60	38,670	2,940,280	1.31%	2	4337/120,479	7133/237,883	Gastric emptying scintillation scan
9	Ji Shangwei^27^	China	January 2011 to December 2013	51.60	51	125	40.80%	2	24,586/1,517,655	14,084/1,422,625	Gastric emptying scintillation scan
10	Qinfei Ye^28^	China	2019–8	59.6	8	61	13.10%	1	0	0	Gastric emptying scintillation scan
11	Qinfei Ye^28^	China	2019–8	63.10	27	456	5.90%	2	0	0	GCSl
12	Chuenyong^29^	Thailand	–	58.92	4	29	13.79%	2	12/268	15/188	GCSl
13	Madeira^30^	Brazil	–	The median age is 28.7 years (22 to 46 years)	5	27	19%	1	0	0	Gastric emptying scintillation scan
14	Madeira^30^	Brazil	–	Median age 57.3 years (47 to 69 years)	11	70	15.70%	2	0	0	GCSI
15	Nicholson^31^	UK	–	46.00	13	115	11.30%	1 and 2	0	0	GCSI
16	Abdulrahmana^32^	Saudi Arabia	–	52.9% Age 40–59 years	29	365	7.90%	1 and 2	0	0	GCSI
17	Gustafsson^33^	Sweden	–	51.3	6	38	15.79%	1	0	0	GCSI
18	Gustafsson^33^	Sweden	–	64.7	5	46	10.87%	2	0	0	Gastric emptying scintillation scan

*The databases searched and the number of studies retrieved are specified as follows: records identified through database searches (PubMed, n = 951; WOS, n = 115; Embase, n = 26; Cochrane Library, n = 25; CNKI, n = 9; WIP, n = 2; WANFANG, n = 3); NPD, Number of patients with DGP (cases); M, Number of males with the disease/total number of males; W, Number of females with the disease/total number of females; D, DGP diagnostic tool.

### Meta-analysis and data statistics

**Figure 2:**
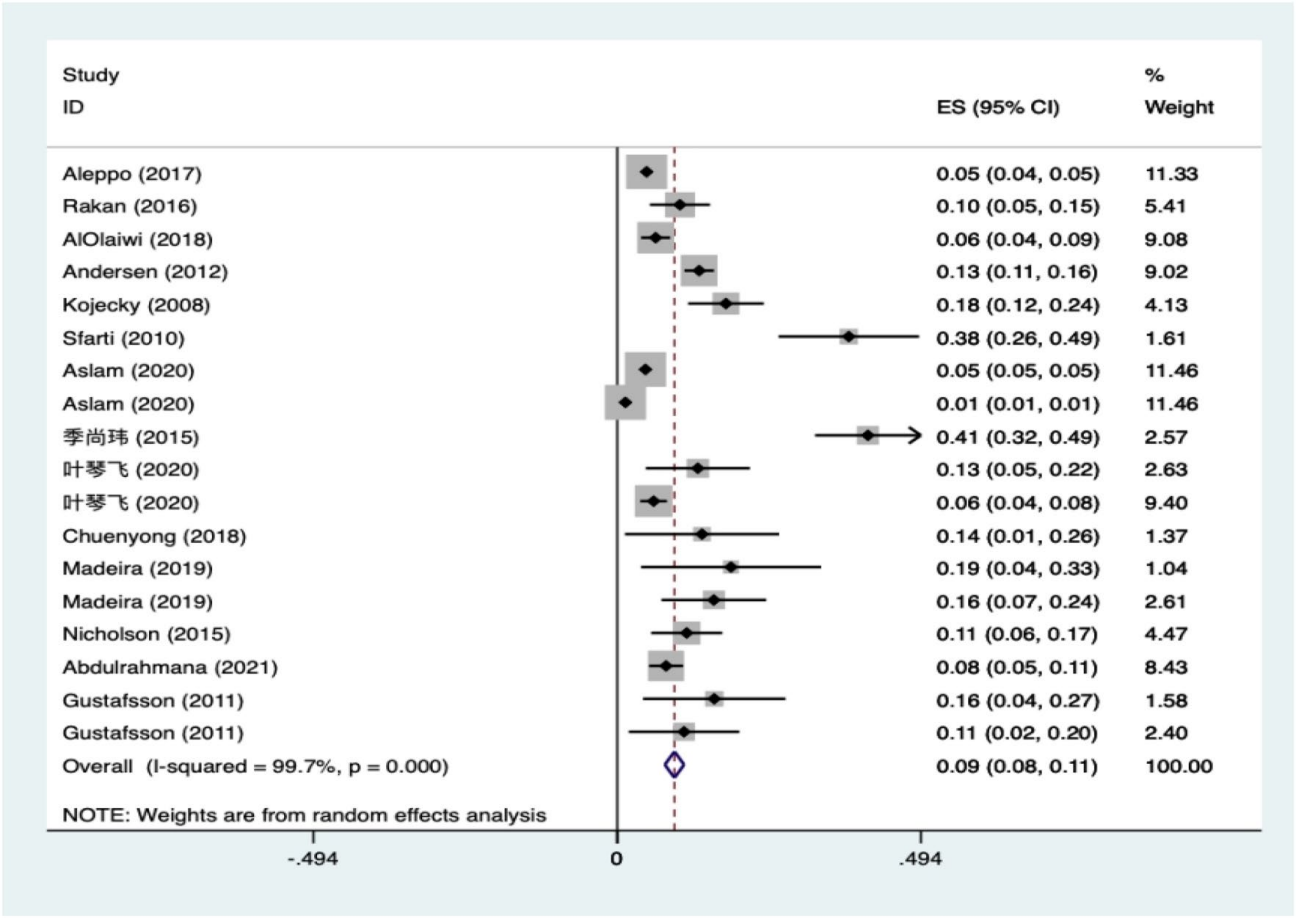
Total prevalence of DM-DGP patients.

A total of 14 studies examined DGP in combination with diabetes in diabetes patients. A heterogeneity test was performed (Fig. 2): χ^2^ = 6428.80, df = 17, *p* < 0.001, with a high level of heterogeneity suggesting a random effects model. The combined presenting prevalence using the D–L method was 9.3%, with a 95% CI ranging from 7.7 to 10.9%, and the meta-analysis revealed a global prevalence of 9.3%. The lowest and highest prevalence, as reported by Aslam et al.^27^, were 1.3% (95% CI 1.3, 1.3) and 40.8% (95% CI 32.2, 49.4), respectively^26,27^. This study’s I^2^ = 99.7% > 50, indicating a high level of heterogeneity between studies and that a funnel plot is not appropriate for assessing publication bias and the Egger test is more accurate in detecting the presence of publication bias.

Subgroup analysis was performed to estimate the prevalence of DGP co-morbid diabetes by sex (excluding some studies that did not examine sex in detail), continent, diabetes typology, method of DGP diagnosis (GCSI and GES), mean age (< 60 or ≥ 60 years), patient source (hospital, clinic, or health care centre), and study method (cross-sectional study, cohort study, or case–control study), as well as to identify potential sources of heterogeneity. A meta-analysis of six screened papers found that women (N = 6, R = 4.6%, 95% CI 3.1%, 6.0%, and I^2^ = 99.8%) had a higher prevalence of DM-DGP than men (N = 6, R = 3.4%, 95% CI 2.0%, 4.7%, and I^2^ = 99.6%). Intergroup *p* = 0.243 > 0.05, suggesting no significant between-group difference in gender (Table 2).

**Table 2:**
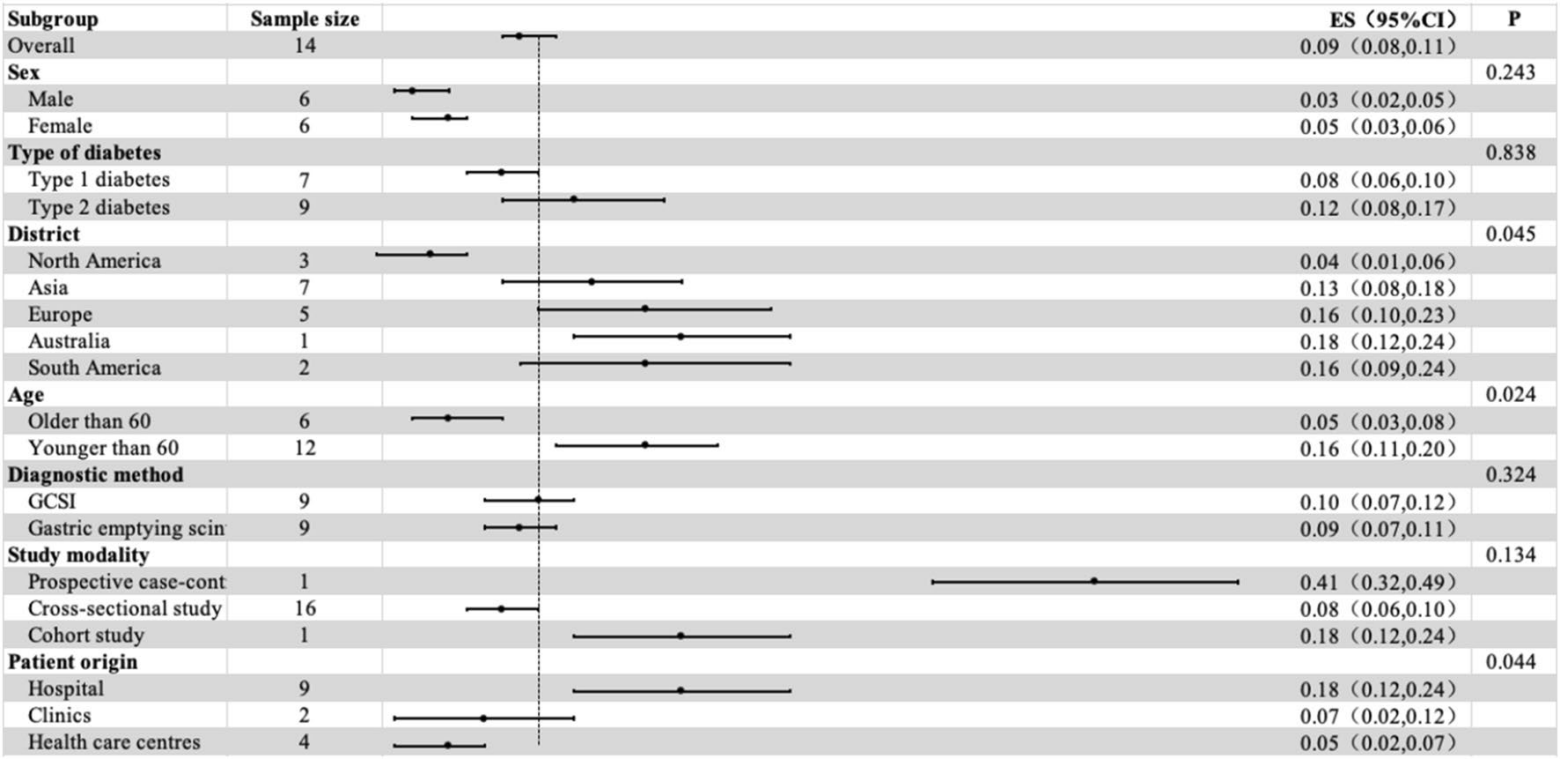
Results of subgroup analysis and differences between groups.

The prevalence of type 2 diabetes (N = 9, R = 12.5%, 95% CI 7.7%, 17.3%, and I^2^ = 95.4%) was higher than that of type 1 diabetes (N = 7, R = 8.3%, 95% CI 6.4%, 10.2%, and I^2^ = 93.6%). Intergroup *p* = 0.838 > 0.05, suggesting that there was not statistically significant between-group difference in the prevalence of type 1 diabetes mellitus and type 2 diabetes mellitus.

Prevalence of DGP by continent. For example, Asia (N = 7, R = 12.6%, 95% CI 7.7%, 17.6%, and I^2^ = 90.7%); Europe (N = 5, R = 16.5%, 95% CI 10.0%, 23.0%, and I^2^ = 77.9%); North America (N = 3, R = 3.6%, 95% CI 1.0%, 6.2%, and I^2^ = 90.7%); South America (N = 2, R = 16.4%, 95% CI 7.7%, 10.9%, I^2^ = 0.0%); and Australia (N = 1, R = 17.7%, 95% CI 11.5%, 23.9%, I^2^ = 0.0%). shows that the prevalence of DGP is higher on all continents except for North America. Intergroup *p* = 0.045 < 0.05, suggesting a statistically significant between-group difference in prevalence by continent.

Furthermore, co-morbid DGP was slightly lower in DM patients aged over 60 years (N = 6, R = 5.5%, 95% CI 3.3%, 7.7%, and I^2^ = 99.9%) compared with those aged under their 60 s (N = 12, R = 15.8%, 95% CI 11.4%, 20.2%, and I^2^ = 88.3%). Intergroup *p* = 0.024 *p* < 0.05 suggests that there is a between group difference in age prevalence between the two groups which is statistically significant.

Subgroup analysis of the prevalence of DGP based on the diagnostic method GCSI: 9.9% (N = 9, 95% CI 7.3%, 12.5%, and I^2^ = 74.5%) was slightly higher than gastric emptying scintigraphy: 8.9% (N = 9, 95% CI 6.7%, 11.2%, and I^2^ = 99.9%). Intergroup *p* = 0.324 > 0.5, suggesting that the two groups were divided by diagnostic method and there was no between-group difference between the two groups, which was not statistically significant.

In the Analyzing subgroups of prevalence based on study modality, the prospective case–control study conducted by Ji Shangwei (2015)^27^ (N = 1, R = 40.8%, 95% CI 32.2%, 49.4%, and I^2^ = 0.0%) was much higher than the cross-sectional study (N = 16, R = 8.0%, 95% CI 6.4%, 9.7%, and I^2^ = 99.8%) and cohort study (N = 1, R = 17.7%, 95% CI 11.5%, 23.9%, and I^2^ = 0.0%). Intergroup *p* = 0.134 > 0.05, suggesting that there is no between-group difference between the three groups, which is not statistically significant.

Subgroup analysis based on patient origin revealed that the prevalence was higher in hospital patients (N = 9, R = 18.4%, 95% CI 12.4%, 24.4%, and I^2^ = 91.8%) than in clinics (N = 2, R = 6.9%, 95% CI 1.7%, 12.1%, and I^2^ = 78.6%) and health care centres (N = 4, R = 4.7%, 95% CI 2.3%, 7.1%, and I^2^ = 99.9%). Intergroup *p* = 0.044 < 0.05, suggesting that there is a statistically significant difference between the three groups based on patient origin.

In the multiple regression model, sample size and publication year heterogeneties were not significant and did not explain the majority of the heterogeneity (*p* > 0.05).

### Publication bias and sensitivity analysis

**Figure 3:**
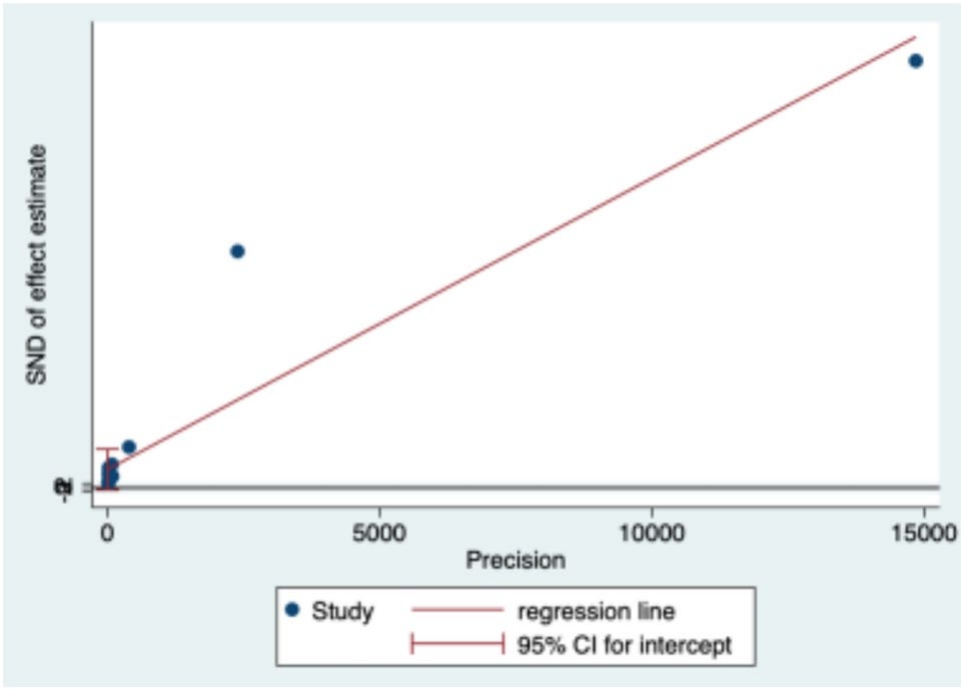
Egger’s test.

**Figure 4:**
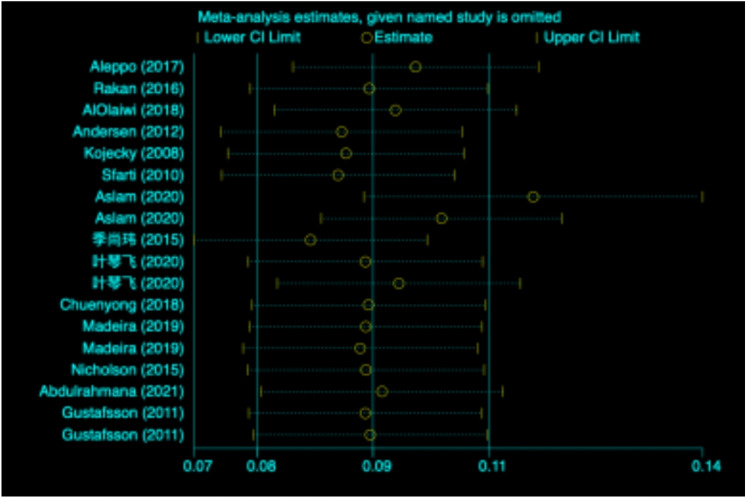
Sensitivity analysis.

Egger’s test (Fig. 3) revealed no significant publication bias (*p* = 0.074). The sensitivity analysis (Fig. 4) revealed that studies were omitted one at a time, with the remaining 14 being combined for meta-analysis. After excluding the study conducted by Aslam et al.^27^, which was considered a source of heterogeneity, resulted in a significant difference between the adjusted and original pooled estimates. After omitting the rest of the studies, the combined results of the remaining studies were found to be statistically significant.

## Conclusion

The meta-analysis revealed that the overall global prevalence of DM-DGP was 9.3%, with a gender difference of 4.6% for women and 3.4% for men. The subgroup analysis revealed that the prevalence of type 2 diabetes was higher than that of type 1 diabetes; the prevalence was higher on all continents except North America, with no significant differences; the prevalence of patients under 60 years of age was significantly higher than that of patients over 60 years of age, indicating an age-related prevalence; and the prevalence of GCSI was slightly higher than that of GES in terms of diagnostic methods for gastroparesis, with no significant differences. In terms of study methods, case–control studies had a much higher relevance than cross-sectional and cohort studies^27^, indicating the impact of the study method on prevalence. However, there was one case–control study, which had a small sample size and was slightly less convincing. Sensitivity analyses suggested the Aslam et al.^27^ study as the main source of heterogeneity. In conclusion, DM-DGP prevalence was associated with gender, diabetes type, age, and method of study.

## Discussion

DGP is a common complication in diabetic patients that is often overlooked in clinical practise. Due to the lack of standardised diagnostic criteria, it is frequently misdiagnosed as a gastrointestinal disorder, leading to inadequate treatment. DM-DGP affects the absorption and metabolism of oral medication, leading to poor glycemic control and further accelerating the course of diabetes, creating a vicious cycle^13^. Therefore, this meta-analysis was conducted to comprehensively analyse the prevalence of DGP in DM globally to help shape healthcare policy. The present study constitutes the first systematic evaluation and meta-analysis on this topic, to our knowledge. Since there was a high degree of heterogeneity in the results, we performed additional correlational subgroup and meta-regression analyses to determine the sources of heterogeneity in terms of year, gender, age, region, diabetes mellitus subtype, and diagnostic method. Although multiple regression analysis revealed no significant differences (*p* > 0.05), subgroup analysis suggested the association of multiple factors. The literature review^34,35^ revealed that the prevalence of co-morbid DM in women is typically higher than in men, which was also consistent with our study. However, we excluded nine papers that did not report accurate data on the prevalence in women or men and retained only five, a small and unconvincing sample size. In addition, several studies have suggested that physical and psychological factors may play a role in diabetes gender difference diabetes. This may therefore have influenced gender differences in DM-DGP patients. Subgroup analysis based on diabetes type indicated a higher prevalence of type 2 diabetes than type 1 diabetes, contradicting some of the literature results^36^. It may be due to the small sample size of type 1 diabetes patients and the high heterogeneity of the type 2 diabetes inclusion study conducted by Aslam et al.^27^, necessitating further research. Previous studies have shown that DGP is a disease associated with aging that is more prevalent in the elderly. Our findings revealed that the prevalence varies with age. One possible explanation is that the definition of age classification in our study was not rigorous and scientific enough. While the majority of the literature uses age mean ± standard error to describe the age status of the study population, we used the mean age as a criterion to classify the age of the patients, where the median age of two papers was also used with some error. It has been suggested in a study^37^ that the prevalence of diabetes increases with age and therefore needs to be investigated further.

The diagnosis of gastroparesis can be challenging and limited by specialised tests. Therefore, early diagnosis of gastroparesis using a gastroparesis symptom score and upper gastrointestinal endoscopy is the first step in ruling out other potential diagnoses. However, the presence of diabetic gastroparesis is suggested by the presence of food residue in the stomach following an overnight fast. Due to the scarcity of resources available for the study of gastroparesis, a barium study may be considered, i.e., little or no gastric emptying of barium after 30 min and complete barium retention after 6 h are indicative of gastroparesis. The 13C-gastric emptying breath test is a reliable diagnostic tool for gastroparesis. However, it has limited utility. According to the American Gastroenterological Association guidelines, GES is a quantitative, non-invasive gastric emptying test using dual radiolabelling and is considered the gold standard test for the diagnosis of gastroparesis. However, the disadvantage of exposing the subject to radiation coupled with the relatively high cost limits its application. This study excluded a large number of non-standard diagnoses during the literature screening, potentially resulting in a smaller sample size and lower prevalence. In terms of literature research methods, case–control and cohort studies had much higher prevalence rates than cross-sectional studies, and only one study existed with an insufficient sample size, which was provisionally considered a heterogeneous source. Based on the source of patients, subgroup analysis was conducted (hospitals, clinics, and health care centers) and revealed a slightly higher prevalence among patients originating from hospitals, which may be attributable to the availability of specialized equipment in hospitals and the high rate of hospital visits by patients.

The limitations of this study include the following: (1) It was not possible to identify adequate factors to account for the observed high degree of heterogeneity through meta-regression and subgroup analyses. Glycaemic control (HbA1C^38^ and FPG within the target levels), metformin use, smoking, alcohol consumption, and lack of exercise may affect the gastrointestinal motility function in patients with T2DM. However, because this information was missing in the included studies, we did not take into account these factors in our study. (2) The diagnostic modalities for DM-DGP are not globally standardized, and GCSI and GES predominate in this study, possibly creating bias between studies. (3) As a result of the small number of included studies (n = 14), the external validity of the results may have been compromised, and we should therefore interpret the findings with caution. (4) Even after sending direct requests to the authors, we could not determine whether males or females were DM-DGP patients in some studies. Therefore, we excluded such studies from the gender subgroup analysis. (5) Some studies failed to report the prevalence of DM-DGP and other crucial data. Therefore, we carried out the analyses using the original data. (6) Statistical methods differed so that the duration of DM could not be analyzed. While some data are expressed as mean ± standard deviation, others are expressed as percentages. Such data made it difficult to perform group analyses. Therefore, future studies are required to investigate diabetes mellitus duration. (7) Egger’s test results with a p-value of less than 0.1 indicate that publication bias may have affected the study results. (8) In this study, we were more concerned with prevalence than incidence. There is no information about whether patients had DGP before developing diabetes, but comorbid conditions are of importance to patients and can lower their quality of life. In the future, we hope to conduct longitudinal studies to investigate the prevalence and risk factors for DGP in patients with DM.

In summary, based on a systematic review and meta-analysis, we present a global prevalence estimate for gastroparesis in adults with diabetes. Taking into account the effects of different study methods in the literature, it was found that the prevalence was highly variable, while other factors did not show significant differences. Furthermore, a high financial and medical burden has been placed on the patient and national medical service with an increased prevalence of DM-DGP patients, national primary health care providers should pay more attention to the status of gastrointestinal motility, long-term glycaemic control, and the prevention and treatment of gastroparesis in DM patients. Not only will prompt and effective treatment of gastroparesis alleviate patients' suffering but it may also improve their glycaemic status.

### Supplementary Information

Supplementary Information.

## Data Availability

The data that support the findings of this study are openly available in [Zhiwang, Wanfang, Wipu, PubMed, Web of Science, Cochrane Library, and Embase].The data on which the study is based were accessed from a repository and are available for downloading through the following link. PubMed:https://pubmed.ncbi.nlm.nih.gov/?term=longqueryf229add0715f271d9686&sort=relevance. Web of Science:https://www.webofscience.com/wos/alldb/summary/b7774276-0e58-43c4-af4e-590a27856eb0-8cd2e5ed/relevance/1. Zhiwang:https://kns.cnki.net/kns8/AdvSearch?dbprefix=CFLS&&crossDbcodes=CJFQ%2CCDMD%2CCIPD%2CCCND%2CCISD%2CSNAD%2CBDZK%2CCCJD%2CCCVD%2CCJFN. Wanfang:https://s.wanfangdata.com.cn/advanced-search/paper. Wipu:http://61.143.209.103:81/Qikan/Search/Advance?from=index. Cochrane Library: https://www.cochranelibrary.com/advanced-search/search-manager. Embase: < https://www-embase-com.heyworld.top/#advancedSearch/resultspage/history.4/page.1/25.items/orderby.date/source.
