# Author Correction: Prevalence of gastroparesis in diabetic patients: a systematic review and meta-analysis

**DOI:** 10.1038/s41598-023-47155-z

**Published:** 2023-11-13

**Authors:** Lianxin Li, Luyao Wang, Ruolan Long, Linrui Song, Rensong Yue

**Affiliations:** Hospital of Chengdu University of Traditional Chinese Medicine, Chengdu, China

Correction to: *Scientific Reports* https://doi.org/10.1038/s41598-023-41112-6, published online 28 August 2023

In the original version of this Article Affiliation 1 was incorrectly given as ‘Department of Endocrinology, Affiliated Hospital of Chengdu University of Chinese Medicine, Chengdu, China’. The correct affiliation is listed below.

Hospital of Chengdu University of Traditional Chinese Medicine, Chengdu, China.

The original Article has been corrected.

