# Supplementary material for: Prevalence of gastroparesis in diabetic patients: a systematic review and meta-analysis
Source: Sci Rep. 2023 Aug 28;13:14015. doi: 10.1038/s41598-023-41112-6 (PMC10462699; doi:10.1038/s41598-023-41112-6)
Supplement: Supplementary file 1 — Supplementary Information. [file 41598_2023_41112_MOESM1_ESM.docx]

**Appendix: Retrieval strategy**

Pubmed

#1糖尿病 757493

(((((((((((((((((((((((((((((((((((((((((((((((((((((("Diabetes Mellitus"[Mesh])) OR (Diabetes Mellitus[Title/Abstract])) OR (Diabetes[Title/Abstract])) OR (Prediabetic State[Title/Abstract])) OR (Glycation End Products, Advanced[Title/Abstract])) OR (Glucose Intolerance[Title/Abstract])) OR (Diabetes Complications[Mesh])) OR (Diabetes Complication[Title/Abstract])) OR (Diabetes-Related Complications[Title/Abstract])) OR (Diabetes Related Complications[Title/Abstract])) OR (Diabetes-Related Complication[Title/Abstract])) OR (Diabetic Complications[Title/Abstract])) OR (Diabetic Complication[Title/Abstract])) OR (Complications of Diabetes Mellitus[Title/Abstract])) OR (Diabetes Mellitus Complication[Title/Abstract])) OR (Diabetes Mellitus Complications[Title/Abstract])) OR ("Diabetes Mellitus, Type 2"[Mesh])) OR (Diabetes Mellitus, Type 2[Title/Abstract])) OR (Diabetes mellitus type 2[Title/Abstract])) OR (type 2 Diabetes mellitus[Title/Abstract])) OR (type 2 Diabetes[Title/Abstract])) OR (Diabetes Mellitus, Noninsulin-Dependent[Title/Abstract])) OR (Diabetes Mellitus, Ketosis-Resistant[Title/Abstract])) OR (Diabetes Mellitus, Ketosis Resistant[Title/Abstract])) OR (Ketosis-Resistant Diabetes Mellitus[Title/Abstract])) OR (Diabetes Mellitus, Non Insulin Dependent[Title/Abstract])) OR (Diabetes Mellitus, Non-Insulin-Dependent[Title/Abstract])) OR (Non-Insulin-Dependent Diabetes Mellitus[Title/Abstract])) OR (Diabetes Mellitus, Stable[Title/Abstract])) OR (Stable Diabetes Mellitus[Title/Abstract])) OR (Diabetes Mellitus, Type II[Title/Abstract])) OR (Diabetes mellitus type II[Title/Abstract])) OR (type II Diabetes mellitus[Title/Abstract])) OR (Maturity Onset Diabetes Mellitus[Title/Abstract])) OR (Diabetes Mellitus, Noninsulin Dependent[Title/Abstract])) OR (Diabetes Mellitus, Maturity-Onset[Title/Abstract])) OR (Diabetes Mellitus, Maturity Onset[Title/Abstract])) OR (Maturity-Onset Diabetes Mellitus[Title/Abstract])) OR (Maturity Onset Diabetes Mellitus[Title/Abstract])) OR (MODY[Title/Abstract])) OR (Diabetes Mellitus, Slow-Onset[Title/Abstract])) OR (Diabetes Mellitus, Slow Onset[Title/Abstract])) OR (Slow-Onset Diabetes Mellitus[Title/Abstract])) OR (Type 2 Diabetes Mellitus[Title/Abstract])) OR (Noninsulin-Dependent Diabetes Mellitus[Title/Abstract])) OR (Noninsulin Dependent Diabetes Mellitus[Title/Abstract])) OR (Maturity-Onset Diabetes[Title/Abstract])) OR (Diabetes, Maturity-Onset[Title/Abstract])) OR (Maturity Onset Diabetes[Title/Abstract])) OR (Type 2 Diabetes[Title/Abstract])) OR (Diabetes, Type 2[Title/Abstract])) OR (Diabetes Mellitus, Adult-Onset[Title/Abstract])) OR (Adult-Onset Diabetes Mellitus[Title/Abstract])) OR (Diabetes Mellitus, Adult Onset[Title/Abstract])

#2 4149

((((((("Gastroparesis"[Mesh])) OR (Gastroparesis[Title/Abstract])) OR (gastroparesis[Title/Abstract])) OR (Gastric Stasis[Title/Abstract])) OR (Gastric Stases[Title/Abstract])) OR (Stases, Gastric[Title/Abstract])) OR (Stasis, Gastric[Title/Abstract])

#3 529148

((((((((((((((((((((((((((("Cross-Sectional Studies"[Mesh])) OR (Cross-Sectional Studies[Title/Abstract])) OR (Cross Sectional Studies[Title/Abstract])) OR (Cross-Sectional Study[Title/Abstract])) OR (Studies, Cross-Sectional[Title/Abstract])) OR (Study, Cross-Sectional[Title/Abstract])) OR (Surveys, Disease Frequency[Title/Abstract])) OR (Disease Frequency Survey[Title/Abstract])) OR (Survey, Disease Frequency[Title/Abstract])) OR (Analysis, Cross-Sectional[Title/Abstract])) OR (Analyses, Cross-Sectional[Title/Abstract])) OR (Analysis, Cross Sectional[Title/Abstract])) OR (Cross-Sectional Analyses[Title/Abstract])) OR (Cross-Sectional Analysis[Title/Abstract])) OR (Cross Sectional Analysis[Title/Abstract])) OR (Analyses, Cross Sectional[Title/Abstract])) OR (Cross Sectional Analyses[Title/Abstract])) OR (Cross-Sectional Survey[Title/Abstract])) OR (Cross Sectional Survey[Title/Abstract])) OR (Cross-Sectional Surveys[Title/Abstract])) OR (Survey, Cross-Sectional[Title/Abstract])) OR (Surveys, Cross-Sectional[Title/Abstract])) OR (Disease Frequency Surveys[Title/Abstract])) OR (Prevalence Studies[Title/Abstract])) OR (Prevalence Study[Title/Abstract])) OR (Studies, Prevalence[Title/Abstract])) OR (Study, Prevalence[Title/Abstract])

#4:#1+#2+#3:948

(((((((((((((((((((((((((((((((((((((((((((((((((((((("Diabetes Mellitus"[Mesh])) OR (Diabetes Mellitus[Title/Abstract])) OR (Diabetes[Title/Abstract])) OR (Prediabetic State[Title/Abstract])) OR (Glycation End Products, Advanced[Title/Abstract])) OR (Glucose Intolerance[Title/Abstract])) OR (Diabetes Complications[Mesh])) OR (Diabetes Complication[Title/Abstract])) OR (Diabetes-Related Complications[Title/Abstract])) OR (Diabetes Related Complications[Title/Abstract])) OR (Diabetes-Related Complication[Title/Abstract])) OR (Diabetic Complications[Title/Abstract])) OR (Diabetic Complication[Title/Abstract])) OR (Complications of Diabetes Mellitus[Title/Abstract])) OR (Diabetes Mellitus Complication[Title/Abstract])) OR (Diabetes Mellitus Complications[Title/Abstract])) OR ("Diabetes Mellitus, Type 2"[Mesh])) OR (Diabetes Mellitus, Type 2[Title/Abstract])) OR (Diabetes mellitus type 2[Title/Abstract])) OR (type 2 Diabetes mellitus[Title/Abstract])) OR (type 2 Diabetes[Title/Abstract])) OR (Diabetes Mellitus, Noninsulin-Dependent[Title/Abstract])) OR (Diabetes Mellitus, Ketosis-Resistant[Title/Abstract])) OR (Diabetes Mellitus, Ketosis Resistant[Title/Abstract])) OR (Ketosis-Resistant Diabetes Mellitus[Title/Abstract])) OR (Diabetes Mellitus, Non Insulin Dependent[Title/Abstract])) OR (Diabetes Mellitus, Non-Insulin-Dependent[Title/Abstract])) OR (Non-Insulin-Dependent Diabetes Mellitus[Title/Abstract])) OR (Diabetes Mellitus, Stable[Title/Abstract])) OR (Stable Diabetes Mellitus[Title/Abstract])) OR (Diabetes Mellitus, Type II[Title/Abstract])) OR (Diabetes mellitus type II[Title/Abstract])) OR (type II Diabetes mellitus[Title/Abstract])) OR (Maturity Onset Diabetes Mellitus[Title/Abstract])) OR (Diabetes Mellitus, Noninsulin Dependent[Title/Abstract])) OR (Diabetes Mellitus, Maturity-Onset[Title/Abstract])) OR (Diabetes Mellitus, Maturity Onset[Title/Abstract])) OR (Maturity-Onset Diabetes Mellitus[Title/Abstract])) OR (Maturity Onset Diabetes Mellitus[Title/Abstract])) OR (MODY[Title/Abstract])) OR (Diabetes Mellitus, Slow-Onset[Title/Abstract])) OR (Diabetes Mellitus, Slow Onset[Title/Abstract])) OR (Slow-Onset Diabetes Mellitus[Title/Abstract])) OR (Type 2 Diabetes Mellitus[Title/Abstract])) OR (Noninsulin-Dependent Diabetes Mellitus[Title/Abstract])) OR (Noninsulin Dependent Diabetes Mellitus[Title/Abstract])) OR (Maturity-Onset Diabetes[Title/Abstract])) OR (Diabetes, Maturity-Onset[Title/Abstract])) OR (Maturity Onset Diabetes[Title/Abstract])) OR (Type 2 Diabetes[Title/Abstract])) OR (Diabetes, Type 2[Title/Abstract])) OR (Diabetes Mellitus, Adult-Onset[Title/Abstract])) OR (Adult-Onset Diabetes Mellitus[Title/Abstract])) OR (Diabetes Mellitus, Adult Onset[Title/Abstract]) AND ((((((("Gastroparesis"[Mesh])) OR (Gastroparesis[Title/Abstract])) OR (gastroparesis[Title/Abstract])) OR (Gastric Stasis[Title/Abstract])) OR (Gastric Stases[Title/Abstract])) OR (Stases, Gastric[Title/Abstract])) OR (Stasis, Gastric[Title/Abstract]) AND ((((((((((((((((((((((((((("Cross-Sectional Studies"[Mesh])) OR (Cross-Sectional Studies[Title/Abstract])) OR (Cross Sectional Studies[Title/Abstract])) OR (Cross-Sectional Study[Title/Abstract])) OR (Studies, Cross-Sectional[Title/Abstract])) OR (Study, Cross-Sectional[Title/Abstract])) OR (Surveys, Disease Frequency[Title/Abstract])) OR (Disease Frequency Survey[Title/Abstract])) OR (Survey, Disease Frequency[Title/Abstract])) OR (Analysis, Cross-Sectional[Title/Abstract])) OR (Analyses, Cross-Sectional[Title/Abstract])) OR (Analysis, Cross Sectional[Title/Abstract])) OR (Cross-Sectional Analyses[Title/Abstract])) OR (Cross-Sectional Analysis[Title/Abstract])) OR (Cross Sectional Analysis[Title/Abstract])) OR (Analyses, Cross Sectional[Title/Abstract])) OR (Cross Sectional Analyses[Title/Abstract])) OR (Cross-Sectional Survey[Title/Abstract])) OR (Cross Sectional Survey[Title/Abstract])) OR (Cross-Sectional Surveys[Title/Abstract])) OR (Survey, Cross-Sectional[Title/Abstract])) OR (Surveys, Cross-Sectional[Title/Abstract])) OR (Disease Frequency Surveys[Title/Abstract])) OR (Prevalence Studies[Title/Abstract])) OR (Prevalence Study[Title/Abstract])) OR (Studies, Prevalence[Title/Abstract])) OR (Study, Prevalence[Title/Abstract])

Wos:155

(TS=(Diabetes Mellitus) OR AB=(Diabetes Mellitus OR Diabetes OR Prediabetic State OR Glycation End Products, Advanced OR Glucose Intolerance) OR TS=(Diabetes Complications) OR AB=(Diabetes Complication OR Diabetes-Related Complications OR Diabetes Related Complications OR Diabetes-Related Complication OR Diabetic Complications OR Diabetic Complication OR Complications of Diabetes Mellitus OR Diabetes Mellitus Complication OR Diabetes Mellitus Complications) OR TS=(Diabetes Mellitus, Type 2) OR AB=(Diabetes Mellitus, Type 2 OR Diabetes mellitus type 2 OR type 2 Diabetes mellitus OR type 2 Diabetes OR Diabetes Mellitus, Noninsulin-Dependent OR Diabetes Mellitus, Ketosis-Resistant OR Diabetes Mellitus, Ketosis Resistant OR Ketosis-Resistant Diabetes Mellitus OR Diabetes Mellitus, Non Insulin Dependent OR Diabetes Mellitus, Non-Insulin-Dependent OR Non-Insulin-Dependent Diabetes Mellitus OR Diabetes Mellitus, Stable OR Stable Diabetes Mellitus OR Diabetes Mellitus, Type II OR Diabetes mellitus type II OR type II Diabetes mellitus OR NIDDM OR NIDDMOR Maturity Onset Diabetes Mellitus OR Diabetes Mellitus, Noninsulin Dependent OR Diabetes Mellitus, Maturity-Onset OR Diabetes Mellitus, Maturity Onset OR Maturity-Onset Diabetes Mellitus OR Maturity Onset Diabetes Mellitus OR MODY OR Diabetes Mellitus, Slow-Onset OR Diabetes Mellitus, Slow Onset OR Slow-Onset Diabetes Mellitus OR Type 2 Diabetes Mellitus OR Noninsulin-Dependent Diabetes Mellitus OR Noninsulin Dependent Diabetes Mellitus OR Maturity-Onset Diabetes OR Diabetes, Maturity-Onset OR Maturity Onset Diabetes OR Type 2 Diabetes OR Diabetes, Type 2 OR Diabetes Mellitus, Adult-Onset OR Adult-Onset Diabetes Mellitus OR Diabetes Mellitus, Adult Onset)) AND (TS=(Gastroparesis) OR AB=(Gastroparesis OR gastroparesis OR Gastropareses OR Gastric Stasis OR Gastric Stases OR Stases, Gastric OR Stasis, Gastric)) AND (TS=(Cross-Sectional Studies) OR AB=(Cross-Sectional Studies OR Cross Sectional Studies OR Cross-Sectional Study OR Studies, Cross-Sectional OR Study, Cross-Sectional OR Surveys, Disease Frequency OR Disease Frequency Survey OR Survey, Disease Frequency OR Analysis, Cross-Sectional OR Analyses, Cross-Sectional OR Analysis, Cross Sectional OR Cross-Sectional Analyses OR Cross-Sectional Analysis OR Cross Sectional Analysis OR Analyses, Cross Sectional OR Cross Sectional Analyses OR Cross-Sectional Survey OR Cross Sectional Survey OR Cross-Sectional Surveys OR Survey, Cross-Sectional OR Surveys, Cross-Sectional OR Disease Frequency Surveys OR Prevalence Studies OR Prevalence Study OR Studies, Prevalence OR Study, Prevalence))

Embase

#1 948911

Diabetes OR Diabetes Mellitus OR Prediabetic State OR Glycation End Products, Advanced OR Glucose Intolerance OR Diabetes Complication OR Diabetes-Related Complications OR Diabetes Related Complications OR Diabetes-Related Complication OR Diabetic Complications OR Diabetic Complication OR Complications of Diabetes Mellitus OR Diabetes Mellitus Complication OR Diabetes Mellitus Complications OR Diabetes Mellitus, Type 2 OR Diabetes mellitus type 2 OR type 2 Diabetes mellitus OR type 2 Diabetes OR Diabetes Mellitus, Noninsulin-Dependent OR Diabetes Mellitus, Ketosis-Resistant OR Diabetes Mellitus, Ketosis Resistant OR Ketosis-Resistant Diabetes Mellitus OR Diabetes Mellitus, Non Insulin Dependent OR Diabetes Mellitus, Non-Insulin-Dependent OR Non-Insulin-Dependent Diabetes Mellitus OR Diabetes Mellitus, Stable OR Stable Diabetes Mellitus OR Diabetes Mellitus, Type II OR Diabetes mellitus type II OR type II Diabetes mellitus OR NIDDM OR NIDDMOR Maturity Onset Diabetes Mellitus OR Diabetes Mellitus, Noninsulin Dependent OR Diabetes Mellitus, Maturity-Onset OR Diabetes Mellitus, Maturity Onset OR Maturity-Onset Diabetes Mellitus OR Maturity Onset Diabetes Mellitus OR MODY OR Diabetes Mellitus, Slow-Onset OR Diabetes Mellitus, Slow Onset OR Slow-Onset Diabetes Mellitus OR Type 2 Diabetes Mellitus OR Noninsulin-Dependent Diabetes Mellitus OR Noninsulin Dependent Diabetes Mellitus OR Maturity-Onset Diabetes OR Diabetes, Maturity-Onset OR Maturity Onset Diabetes OR Type 2 Diabetes OR Diabetes, Type 2 OR Diabetes Mellitus, Adult-Onset OR Adult-Onset Diabetes Mellitus OR Diabetes Mellitus, Adult Onset

#2 6689

Gastroparesis OR gastroparesis OR Gastropareses OR Gastric Stasis OR Gastric Stases OR Stases, Gastric OR Stasis, Gastric

#3 393402

Cross-Sectional Studies OR Cross Sectional Studies OR Cross-Sectional Study OR Studies, Cross-Sectional OR Study, Cross-Sectional OR Surveys, Disease Frequency OR Disease Frequency Survey OR Survey, Disease Frequency OR Analysis, Cross-Sectional OR Analyses, Cross-Sectional OR Analysis, Cross Sectional OR Cross-Sectional Analyses OR Cross-Sectional Analysis OR Cross Sectional Analysis OR Analyses, Cross Sectional OR Cross Sectional Analyses OR Cross-Sectional Survey OR Cross Sectional Survey OR Cross-Sectional Surveys OR Survey, Cross-Sectional OR Surveys, Cross-Sectional OR Disease Frequency Surveys OR Prevalence Studies OR Prevalence Study OR Studies, Prevalence OR Study, Prevalence

4#:#1+#2+#3=26

Cochrane library:25
